# Continuing Professional Development—Medical Imaging

**DOI:** 10.1002/jmrs.70081

**Published:** 2026-03-15

Maximise your continuing professional development (CPD) by reading the selected article and answering the five questions. Please remember to self‐claim your CPD and retain your supporting evidence. Answers will be available via the QR code and published in JMRS—Volume 73, Issue 4, December 2026.

## Proposed Diagnostic Reference Levels for Frequently Performed Paediatric Radiographic Examinations

Edel Doyle, Matthew R. Dimmock, Kam L. Lee, Peter Thomas, Richard B. Bassed, https://doi.org/10.1002/jmrs.866.

1. According to the Australian Radiation Protection and Nuclear Safety Agency (ARPANSA), what is the primary purpose of Diagnostic Reference Levels (DRLs) in medical imaging?
  - To set mandatory dose limits for all patients
  - To prompt investigation if consistently exceeded (or too low) for standard imaging examinations
  - To determine the cost of radiographic procedures
  - To replace individual patient dose assessments
2. According to the International Commission on Radiological Protection (ICRP), which patient characteristic is preferred for stratifying paediatric DRLs?
  - Age
  - Height
  - Weight
  - Gender
3. What metric was used in this study to determine radiation dose for establishing DRLs in paediatric radiography?
  - Entrance Surface Dose (ESD)
  - Dose Area Product (DAP)
  - Air Kerma Area Product (KAP)
  - Effective Dose
4. In this Australian study, which body part was found to be the most radiographed in children under 80 kg?
  - Abdomen
  - Chest
  - Wrist
  - Foot
5. When establishing a Facility Reference Level (FRL), what is the recommended minimum number of patients required to ensure data reliability?
  - 3
  - 5
  - 10
  - 20

### Recommended Further Reading

1. T. Zaidi and R. Nezich. “Radiation Dose and Risk in the Radiological Investigation of Suspected Non‐Accidental Injury (NAI).” *Journal of Medical Radiation Sciences* (2025): published ahead of print, December 4, 2025, https://doi.org/10.1002/jmrs.70045.
2. S. M. Lilli and A. A. Perdomo. “Establishing Facility Reference Levels and Comparing to International Diagnostic Reference Levels in Paediatric General X‐Ray.” *Physical and Engineering Sciences in Medicine* 48, no. 3 (2025): 1015–1022, https://doi.org/10.1007/s13246‐025‐01563‐9.
3. S. Sait, H. G. Newman, S. Das, and S. Haque. “Effective Radiation Dose of Skeletal Surveys Performed for Suspected Physical Abuse.” *Pediatric Radiology* 53, no. 1 (2023): 69–77, https://doi.org/10.1007/s00247‐022‐05477‐6.

## Answers

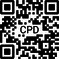

Scan this QR code to find the answers.

